# Model-Based Insights into Multi-Host Transmission and Control of Schistosomiasis

**DOI:** 10.1371/journal.pmed.0050023

**Published:** 2008-01-22

**Authors:** Song Liang, Robert C Spear

## Abstract

The authors discuss a new study that helps to elucidate the determinants of schistosomiasis transmission in an endemic area of the Philippines.

Schistosomiasis is a tropical disease of great antiquity that remains endemic in 76 countries, affecting 200 to 300 million people in the developing world. Current control of schistosomiasis is heavily dependent on chemotherapy, primarily through praziquantel, a safe and highly effective drug introduced in the early 1980s. In recent decades, large-scale school- or community-based treatment strategies have been successful in suppressing average infection intensity in many parts of the world, dramatically reducing schistosomiasis-associated morbidities such as hepatosplenomegaly, hepatic fibrosis, or bladder and kidney inflammation for urinary schistosomiasis [1,2]. We expect to see a similar pattern in low-income countries, particularly in sub-Saharan Africa, as praziquantel is made increasingly available [3].

However, increasing evidence shows that chemotherapy-based strategies alone are unlikely to be a sustainable strategy for prevention of schistosome infections in all endemic areas [2,4,5]. This evidence has two important implications. First, although advanced stages of schistosomiasis can be effectively controlled through praziquantel use, there is evidence that light chronic infections due to continuing transmission contribute significantly to chronic morbidities such as growth retardation, anemia, exercise intolerance, poor school performance, and lower work capacity [6]. Second, drug treatment does not change the environmental conditions that foster transmission of the parasite in endemic communities [2,4]. In some environments, the cessation of treatment even for a few years can result in a re-occurrence of high levels of infection, even back to pretreatment levels in extreme cases [7,8]. Hence, there is a pressing need to return to a more comprehensive strategy for suppressing schistosomiasis transmission beyond drug treatment.

**Linked Research Article**

This Perspective discusses the following new study published in *PLoS Medicine:*

Riley S, Carabin H, Belisle P, Joseph L, Tallo V, et al. (2008) Multi-host transmission dynamics of Schistosoma japonicum in Samar Province, the Philippines. PLoS Med 5(1): e18. doi:10.1371/journal.pmed.0050018

Obtaining schistosome infection data from thousands of humans and mammalian hosts in the Philippines, Steven Riley and colleagues find that mammalian acquisition, rather than transmission to snails, drives prevalence.

In the continuing absence of viable vaccines against the schistosome, there is reason to reconsider earlier approaches targeting the parasite transmission cycle to augment the chemotherapy-based strategy. However, such approaches (e.g., mollusciciding, improved sanitation, and provision of safe water) require significantly greater financial resources than does chemotherapy. To make best use of limited resources, the key is to identify and target control efforts at locally vulnerable stages of the transmission cycle. Although the complete life cycle of the schistosome was described almost a century ago, characterizing local or regional vulnerabilities of the parasite-snail-host interaction still remains a challenging task because of its complex dependence on agricultural and other ecological factors. This is particularly true for Schistosoma japonicum, which, in contrast to other species of schistosome, has a number of nonhuman mammalian hosts. The contribution of these zoonotic carriers to transmission is seldom well characterized [9]. In a new study in *PLoS Medicine*, Steven Riley and colleagues present an analysis using a mathematical model complemented with statistical approaches to unravel this particular aspect of the transmission cycle in an endemic region in the Philippines [10].

## Using a Mathematical Model as a Platform for Synthesis and Interpretation of Field Data

Mathematical models have long been recognized as useful tools in exploring complicated relationships underlying infectious disease transmission processes. Usually, the structure of the model is based on a set of causal hypotheses that describe current understanding of how different processes are interrelated [11]. Unlike statistical models, their parameters generally have physical or biological meaning that allows their values to be estimated from literature as well as from field and experimental data. Like statistical models, mathematical models can be used to test competing hypotheses underlying research questions.

Riley and colleagues' study was based on a relatively large-scale cross-sectional survey of S. japonicum in 50 villages in the Province of Samar, the Philippines, where stool samples from 5,623 humans and 5,899 potential nonhuman mammalian hosts (dogs, cats, pigs, water buffalo, and rats) were examined. The detection and quantification of schistosome eggs in stool is subject to low sensitivity and non-optimal specificity. The authors acknowledge this limitation in their study [10].

Specifically, they assigned humans into three infection classes (none, light, and heavy) and the animals into two (uninfected and infected). They then built a conceptually straightforward model to track the transmission cycle from mammals to snails and back from snails to each category of mammalian host. The authors estimated transmission parameters for the 50 villages based on three hypotheses: H_o_, transmission rates (snails to mammalian hosts and mammalian hosts to snails) are constant for all villages; H_1_, transmission rates from mammalian hosts to snails are site-specific, varying by village; and H_2_, transmission rates from snails to mammalian hosts are site-specific by village. The goodness of fit of the model was then evaluated under each of the three scenarios. Based on these comparisons, inferences were made subsequently with regard to relative roles of different hosts and transmission stages in the villages.

## Key Findings

For hypothesis H_0_, the model produced a poor fit to the observed variations in the adjusted data, suggesting that it is not the distribution of potential mammalian reservoir and human hosts in each village that drives the village-to-village variations in transmission. Assuming transmission from mammalian hosts to snails to be site-specific (H_1_) produced a better fit to human infection data. However, allowing the rate of transmission from snails to mammalian hosts to vary by village (H_2_) resulted in a substantial improvement in explaining trends in human infection.

Not surprisingly, humans were found to be more susceptible to infection than any other species of nonhuman hosts. Of particular interest is that, for the nonhuman mammalian hosts, buffalo (which have the largest body sizes and amount of daily fecal output among the all nonhuman hosts) and dogs play a marginal role in transmission, while rats (which have the smallest body sizes and fecal output) may be important.

## Study Limitations and Public Health Implications

This research represents a useful contribution to elucidating the determinants of schistosomiasis transmission in an endemic area of the Philippines, and it illustrates the use of a mathematical model, complemented with statistical approaches, in exploring the roles of multiple mammalian hosts. However, the study is subject to several limitations. Most notably, and as acknowledged by the authors, is the exclusion of demographic characteristics such as age, gender, and occupation of the human population. These factors have been shown to be important risk factors of schistosomiasis in a number of studies.

Second, there is essentially no village-level environmental data included in the analysis. In particular, the assumption of constant snail density for all villages precludes the assessment of the impact of differences in snail populations in these villages. Although the dependence of transmission intensity on snail density and their within-village distribution is unclear, snail dynamics have been shown to be important to local transmission of S. japonica in irrigated villages in China, as have other environmental factors [12]. Similarly, the suggestion that rats may play an important role in transmission is weakened by the lack of information on rat population sizes.

So, the most robust outcomes of the model-based analyses are qualitative: that the village is an important determinant of transmission intensity and that animal populations are of secondary importance in sustaining endemic levels of transmission. Further, it seems likely that the village-level determinants are related to snail populations, with the implication that interventions aimed at suppression of snail population, thereby reducing mammalian exposures, may be more effective than targeting other parts of the transmission cycle. These findings, however, may not all be generalizable to other settings—for example, buffalo may play an insignificant role in transmission in the Philippine villages, but they have been shown to contribute substantially to transmission in many parts of China. The particular strategic value of this study is the inferential framework it exemplifies. The modeling approach can be a useful tool in exploring schistosomiasis transmission in other settings, and may even apply to other macroparasites.
